# Rare SRY-negative 46,XX disorder of sex development with male phenotype and ectopic gonads: a case report

**DOI:** 10.3389/fendo.2026.1829751

**Published:** 2026-04-30

**Authors:** Jianxu Luo, Fuxin Huang, Jianlin Li, Enhao Mo, Hu Wang, Jianyong Zhang, Caifeng Pang, Dezheng Lei, Jiabo Chen

**Affiliations:** 1 Department of General Surgery, Maternal and Child Health Hospital of Guangxi Zhuang Autonomous Region, Nanning, China; 2 Department of Urology, Maternal and Child Health Hospital of Guangxi Zhuang Autonomous Region, Nanning, China

**Keywords:** 46,XX male, case report, disorder of sex development, ectopic gonads, SRY-negative

## Abstract

Individuals who exhibit male external genitalia and testicular tissue despite a 46,XX karyotype with SRY-Negative represent an exceptionally rare phenotype of disorders of sex development (DSD), and the underlying mechanism remains poorly understood. We report an 18-year-old patient with a 46,XX karyotype and a male phenotype who presented with a 2-year history of left scrotal pain. Physical examination demonstrated male-type external genitalia. Imaging revealed a uterine-like structure within the left hemiscrotum with intracavitary blood accumulation suggestive of retained menstrual blood, and testicular-like tissue in the right hemiscrotum; these findings were subsequently confirmed by surgical exploration and histopathology. In addition, ovarian tissue structures were also identified in the pathological specimens. Endocrine evaluation showed abnormally elevated serum levels of luteinizing hormone and estradiol, with abnormally decreased of testosterone. Our report highlights the diagnostic and therapeutic challenges of SRY-negative 46,XX DSD with a male phenotype and provides clinically relevant insights for differential diagnosis, pathogenetic considerations, and individualized management.

## 1 Introduction

Disorders of sex development (DSD) refer to a group of congenital conditions characterized by discordance among chromosomal, gonadal, and phenotypic sex, commonly presenting with external genital anomalies, gonadal dysfunction, and reproductive system disorders (1). Clinical management of DSD remains highly challenging due to its complex etiology involving genetic regulation, psychosexual development, and social environmental factors, as well as marked heterogeneity. Notably, rare subtypes are prone to diagnostic delays attributed to atypical manifestations and limited clinical experience.

46,XX DSD with male phenotype is an extremely rare subtype of DSD with an incidence of approximately 1∶20,000 (2). The pathogenesis in SRY-negative individuals differs from the classic sex development theory proposed by Alfred Jost—lacking the Y-chromosomal SRY gene, this subtype drives primordial gonadal differentiation into testes independently via abnormal expression or mutations of downstream genes such as SOX9 and SOX3 (3). Currently, there is no unified clinical guideline for this condition, and the understanding of its phenotypic characteristics, genetic spectrum, and prognosis remains limited.

To accumulate clinical experience of rare cases and optimize diagnostic and therapeutic strategies, this study details a case of 46,XX DSD with male phenotype and ectopic gonads and Müllerian structures. By analyzing clinical features, laboratory findings, and genetic testing results, we discuss the underlying pathogenesis and key clinical management points, aiming to provide references for the early identification and precise intervention of similar diseases in clinical practice.

## 2 Case report

An 18-year-old phenotypic male was admitted to our department with a 2-year history of intermittent left scrotal pain. At birth, the patient presented with ambiguous genitalia consistent with Prader stage IV, featuring penile curvature and abnormal urethral meatus, with testis-like structures palpable bilaterally in the scrotum. A clinical diagnosis of hypospadios was established, and surgical repair was performed at the age of 3 years with an uneventful postoperative course. The patient was reared as a male from birth. Intermittent left scrotal pain developed 2 years prior to presentation, with monthly episodes lasting 4 to 7 days. Progressive enlargement of the left hemiscrotum was identified, while the right hemiscrotum exhibited no significant changes in size or associated symptoms. No family history of similar disorders was documented among first-degree relatives.

**Figure 1:**
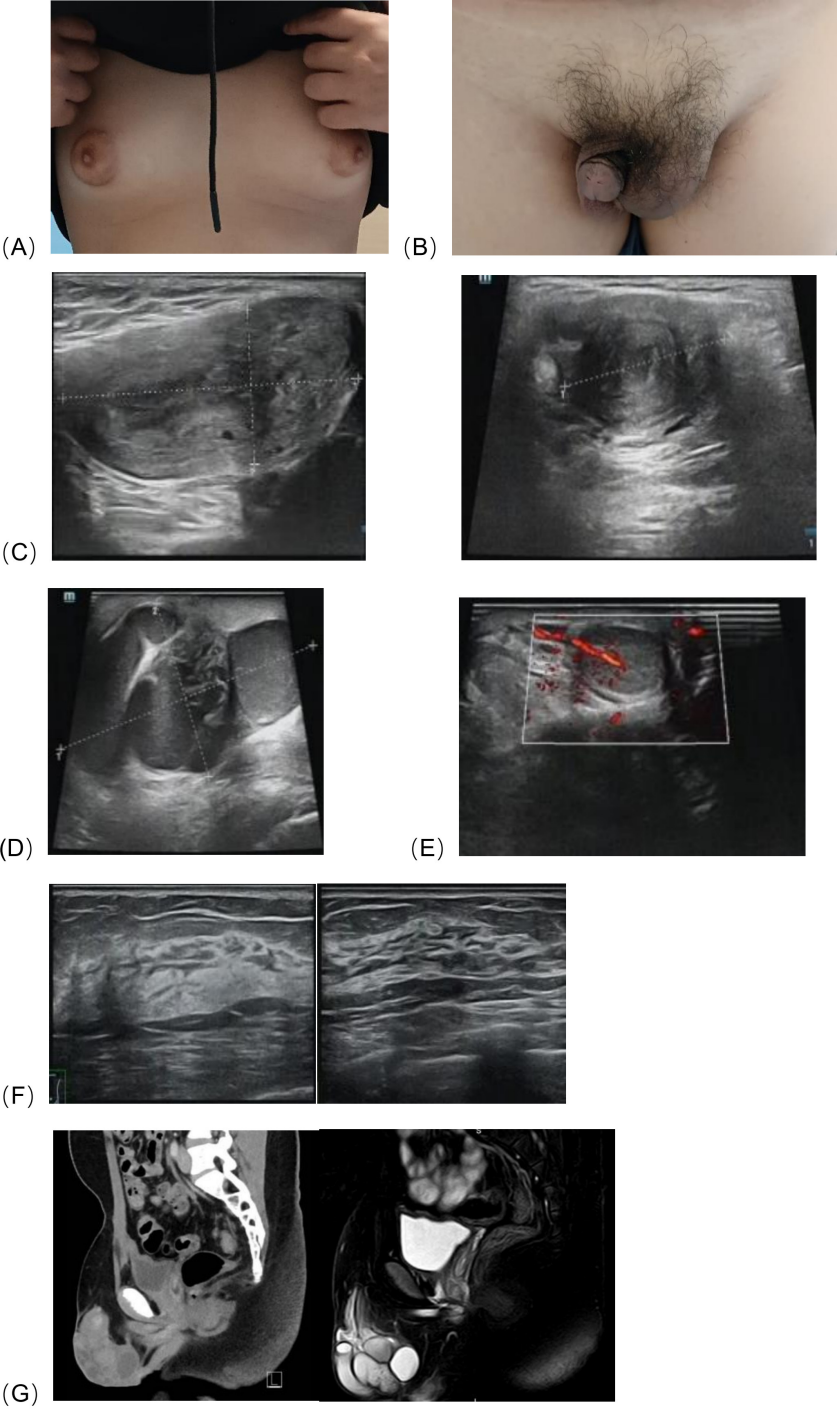
**(A)** Bilateral breast development, **(B)** The patient’s external genitalia, **(C)** Ultrasonography revealed an abnormal solid mass in the left inguinal region and scrotum, consistent with a uterus, measuring approximately 5.7×3.0×2.4 cm, with endometrial echo (thickness ~0.7 cm) and uterine cavity separation (~0.4 cm). **(D)**Surrounding the mass were tortuous, dilated tubular anechoic areas, suggestive of fallopian tubes, measuring approximately 7.1×4.2×6.1 cm. **(E)** The right scrotum contained testicular parenchyma, measuring 2.0×1.4×0.8 cm. **(F)** Breast ultrasound showed glandular tissue in both breasts, measuring 1.2 cm in anteroposterior diameter, with no detectable masses. **(G)** CT and MRI revealed a tubular soft tissue structure with a lumen, measuring approximately 4.8×1.5×1.1 cm, was detected between the bladder and rectum.

On physical examination, the patient’s vital signs were as follows: temperature 36.5 °C, blood pressure 112/65 mmHg, height 158 cm, weight 66.4 kg, and BMI 26.6 kg/m². The chest was symmetrical, and no abnormalities were noted. Cardiopulmonary and abdominal examinations were unremarkable. Bilateral breast development was observed (**Figure 1A**), consistent with Tanner stage III. The external genitalia exhibited a male phenotypic appearance; pubic hair was sparse and limited in distribution, corresponding to Tanner stage III. The flaccid penile length was 2.5 cm (reference range: 7.3 ± 1.0 cm), stretched penile length was 4.0 cm (reference range: 13.1 ± 1.1 cm), penile circumference was approximately 3.5 cm (reference range: 8.1 ± 0.6 cm), and erectile length was not measured (**Figure 1B**). The urethral meatus located approximately 0.5 cm below the glans. Scarring from the previous hypospadias surgery was evident on the ventral side of the penis. On examination of the scrotum, The right hemiscrotum was normal in morphology and size, without ectopic tissue or cryptorchidism. A testis-like structure measuring approximately 1.0 cm × 0.6 cm × 0.5 cm was palpable in the right hemiscrotum, with medium consistency, good mobility, and no tenderness. The left hemiscrotum was significantly enlarged, containing a solid nodular mass measuring approximately 6.0 cm × 5.0 cm × 3.0 cm, which was hard in consistency, non-tender, and irreducible. No definite gonadal tissue was palpable in the left hemiscrotum. No edema, fistula, or other abnormalities were observed on the scrotal skin bilaterally. No vaginal opening was found in the perineum.

Ultrasonographic examination of the scrotum and testes revealed an abnormal solid mass in the left hemiscrotum, which was suggestive of a uterus with possible intrauterine fluid accumulation, measuring approximately 5.7×3.0×2.4 cm (**Figure 1C**). Above the left hemiscrotum and in the left inguinal region, a tortuous, dilated tubular hypoechoic area was observed, with an irregular shape and approximate dimensions of 7.1×4.2×6.1 cm, raising the possibility of hydrosalpinx (**Figure 1D**). Testicular tissue, measuring about 2.0×1.4×0.8 cm, was identified in the right hemiscrotum (**Figure 1E**). No ovarian tissue was detected in the pelvic cavity. Ultrasonography of the adrenal glands, liver, gallbladder, pancreas, spleen, and kidneys showed no abnormalities. Glandular structures were noted in both breast regions (**Figure 1F**). Subsequent CT and MRI examinations revealed no gonadal tissue in the pelvic or abdominal cavity; however, a tubular soft tissue structure with a lumen was observed between the bladder and rectum, with unclear characteristics, measuring approximately 4.8×1.5×1.1 cm (**Figure 1G**). Laboratory tests, including complete blood count, urinalysis, stool analysis, and metabolic panel, were all within normal limits. Tumor markers were also within the normal range. Endocrine analysis showed elevated levels of luteinizing hormone and estradiol, together with decreased testosterone levels, as detailed in the **Table 1**.

**Table 1:** Hormone levels of the patient.

Indicators	Laboratory data	Reference values
FSH	11.14 IU/l	1.5–12.4 IU/L
LH	13.49 IU/l	0.56-5.96 IU/L
PRL	185.70 mIU/L	102–264 mIU/L
Estradiol	221.40 pmol/L	36.7–146.8 pmol/L
Progesterone	0.52 nmol/L	0.27–0.94 nmol/L
Testosterone	1.71 nmol/L	8.64–29.0 nmol/L
Aldosterone	101.43 pg/ml	40–310 pg/ml
17α-OHP	2.13 nmol/L	0.4–3.0 nmol/L
DHEA-S	251 μg/dl	100–400 μg/dl
DHT	117.26 pg/ml	30–180 pg/ml
ACTH	3.24 pmol/L	1.6–13.9 pmol/L

**Figure 2:**
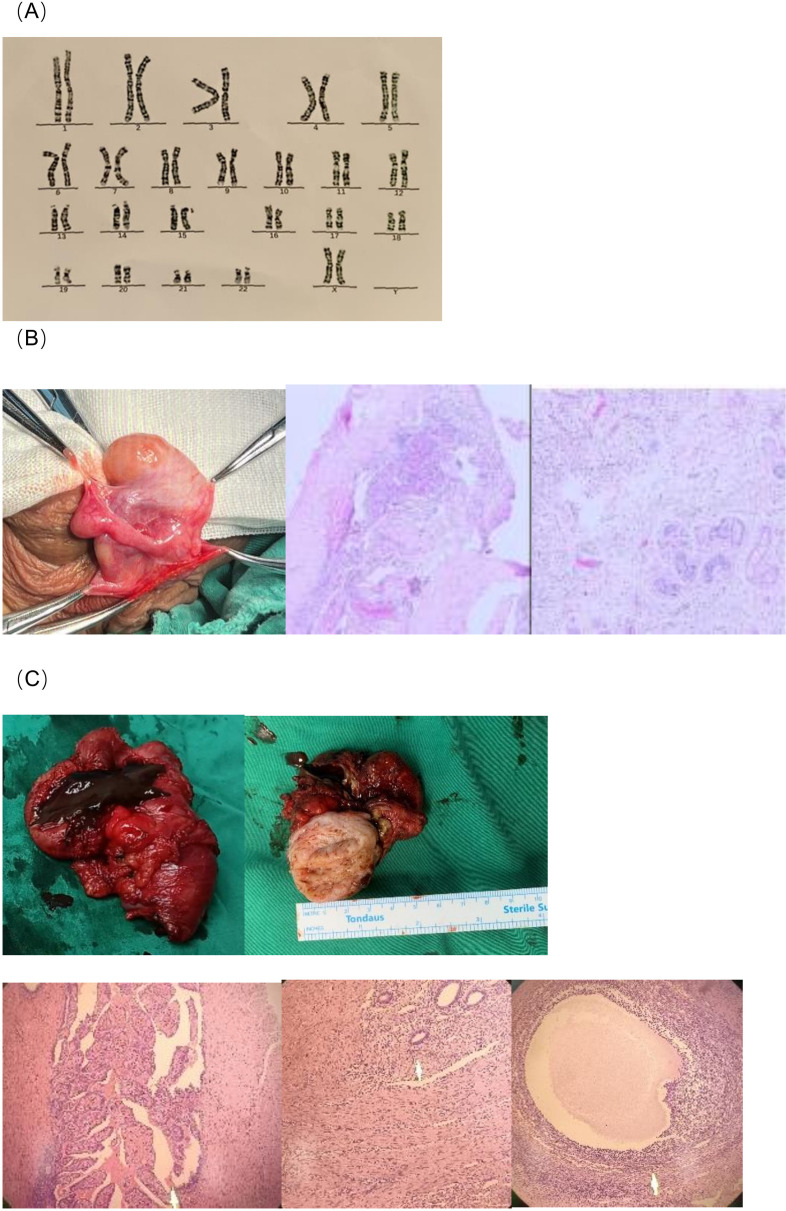
**(A)** Chromosome analysis using G-banding revealed a karyotype of 46, XX. **(B)** The right gonadal biopsy was consistent with testis. Microscopic description:Several seminiferous tubules were identified, containing Sertoli cells. There was maturation arrest of spermatogenic cells, and no spermatozoa were identified. **(C)** Gross description:Uterine-like structure and fallopian tube-like structures were densely adherent, measuring approximately 8×5×4 cm. The fallopian tube-like structures were tortuous and dilated, containing old blood on sectioning. A uterine cavity was identified upon sectioning the uterine-like structure. Ovarian parenchyma was not grossly identified. Microscopic description:The uterine-like structure consisted of smooth muscle, with endometrial glands and stroma present on the surface and within the parenchyma. Ovarian stroma, follicles, and fallopian tube structures were identified. The fallopian tube showed features consistent with xanthogranulomatous salpingitis.

Genetic analyses were performed using genomic DNA extracted from the patient’s peripheral blood sample. High-throughput sequencing was used to perform whole-exome sequencing (WES). Combined with the multi-center screening process for pathogenic variants associated with sex differentiation abnormalities, no pathogenic variants that were highly correlated with the patient’s definite clinical phenotype and supported by sufficient pathogenic evidence were detected. Chromosome karyotype analysis was performed using G-banding technique, and the results showed a karyotype of 46, XX (**Figure 2A**); the SRY gene test result in this sample was negative.

Diagnostic laparoscopy and management were performed under general anesthesia, including laparoscopic exploration and bilateral gonadal biopsy. Intraoperative exploration identified testicular tissue in the right hemiscrotum (**Figure 2B**), and the right vas deferens was visualized in the pelvic/abdominal cavity as well as in the right hemiscrotum, while no gonadal tissue was identified on the left hemiscrotum. No soft tissue structure was found in the rectovesical pouch during exploration. Histological examination of the gonadal tissue samples from the right hemiscrotum, after formalin fixation, paraffin embedding, and hematoxylin-eosin staining, showed multiple seminiferous tubules interspersed within fibrous tissue, with thickened and fibrotic walls. Support cells were observed within the tubules, but spermatogenesis was absent, and no sperm were identified (**Figure 2B**).

Based on the biopsy results, we engaged in multiple discussions with the patient. The patient expressed a strong desire to retain male-associated gonads and sexual characteristics, but had no intention of reproduction. Following thorough informed consent, psychological evaluation, and approval by the ethics committee, we decided to proceed with the complete excision of the left scrotal mass while preserving the right testis and penis. Postoperatively, the patient recovered well. The pathological and histological findings of the left scrotal mass are shown in **Figure 2C**.

## 3 Discussion

46,XX DSD with male phenotype, is an extremely rare DSD. It is characterized by varying degrees of masculinization in sexual characteristics, behavior, growth and development, and skeletal development, despite the absence of the Y chromosome. The syndrome was first reported by De la Chapelle et al. in 1964 (4), with an incidence of approximately 1∶20,000–25,000 in newborn male infants, whereas ovotesticular DSD has been reported in some studies to have an incidence as low as 1:100,000 live births (5). Its pathogenesis is highly complex and has not yet been fully elucidated. While genetic alterations involving the Y chromosome are well-established drivers of 46,XX testicular DSD, the dysregulated balance within the molecular network governing sex determination represents a critical complementary pathogenic basis, especially in SRY-negative patients (6). Aberrant activation of the testis-differentiation pathway stands as a core event in this context, wherein dysregulation of SOX9 is the most prevalent molecular etiology (7, 8). Specifically, copy number variations or gain-of-function mutations in the SOX9 coding sequence or its upstream enhancer regions, together with functional compensation by SOX family homologs (e.g., SOX3 and SOX10), enable SOX9 to circumvent the SRY-dependent activation machinery, culminating in aberrant overexpression that triggers testicular differentiation (9). Concurrently, suppression of the ovary-differentiation pathway via loss-of-function mutations in *RSPO1*, *WNT4*, or *NR5A1*—or functional impairment of *FOXL2* that induces transdifferentiation of ovarian somatic cells into Sertoli-like cells—creates a permissive microenvironment for masculinization (10–12). In contrast, the canonical genetic mechanisms involve direct acquisition or mosaic presence of Y-chromosomal determinants. Xp-Yp translocation introduces Y-chromosomal genetic material into the 46,XX genome, leading to ectopic expression of testis-determining factors9. Similarly, 46,XX/46,XY mosaicism underpins masculinization in a subset of cases, whereby the presence of a minor SRY-positive cell line is sufficient to skew gonadal development toward testicular differentiation (13, 14).

In this study, we performed a systematic review of reported cases of 46,XX male reversal syndrome (**Tables 2**, **3**). The SRY gene is critical for male sex determination, and its detection holds great significance for the clinical diagnosis of sex reversal syndrome. In addition to whole-exome sequencing (WES), the detection of the SRY gene across multiple tissues using fluorescence *in situ* hybridization (FISH) and polymerase chain reaction (PCR) also represents an important approach (15). Most patients with 46,XX testicular DSD are SRY−positive, as the SRY gene directly drives male gonadal and phenotypic differentiation (15). Such individuals usually present with a normal male phenotype and often seek medical attention due to sterility, accompanied by clinical manifestations including hypergonadotropic hypogonadism. In contrast, SRY−negative 46,XX DSD patients typically exhibit atypical masculinization (16). Most are born with ambiguous genitalia, and female secondary sexual characteristics such as breast development may even occur in adult patients, whose gonads are more frequently ovotesticular (17, 18). The patient in the present case displayed a predominantly male phenotype, but also presented with breast development (Tanner stage III) and cyclical menstrual−related symptoms (cyclical pain in the left intrascrotal mass), suggesting the presence of functional ovarian tissue. Limited by the resolution and localization constraints of imaging examinations, ovarian structures were not clearly identified preoperatively. However, intraoperative and histopathological findings confirmed that, apart from a tubular structure between the bladder and rectum (Müllerian derivative), the patient’s Müllerian derivatives, including the uterus and fallopian tubes, as well as the ovarian tissue, were not located in the abdominal cavity, but were ectopically positioned in the left scrotum. Histopathological examination of the right hemiscrotum confirmed the presence of a dysplastic testis, consistent with the patient’s decreased testosterone levels. To the best of our knowledge, as far as the literature is concerned, this presentation has not been previously documented. Based on laparoscopic findings, we hypothesize that this abnormality may have resulted from herniation of the Müllerian derivatives and ovary into the scrotum via the inguinal canal during embryonic or postnatal development.

**Table 2:** Clinical, genetic, hormonal and therapeutic characteristics of patients with 46,XX DSD male phenotype.

Reference	Gender	Age (y)	Chef complaints	Karyotype (peripheral leukocytes)	Gene mutations	SRY	Treatment	Ambiguous genitalia (prader)
Ruben Lisker et al (1970) (19)	M	37	sterility	46XX	NA	NA	NA	+ (5)
Sarah F Slaney et al (1998) (17)	M	10	ambiguous genitalia	46XX	NA	–	gonad resection+testosterone replacement	+ (4)
Jianhong Li,Tianhua Huang et al (2004) (20)	M	2-7 (n=5)	Hypospadias	46XX	NA	+ (n=3) NA (n=2)	hypospadias repair	+ (4)
T. Wang et al. (2009) (15)	M	20	undescended testicles (right)	46XX	NA	+	NA	+ (NA)
Xuefeng Gao et al (2012) (21)	M	adult age	sterility	46XX (n=11)	NA	+ (n=10) - (n=1)	NA	NA
Annalisa Vetro et al (2015) (18)	M	adult age	sterility	46XX	SOX9	–	NA	+ (NA)
Kangying Wang et al (2018) (22)	M	NA	sterility	46XX	NA	+	NA	+ (5)
Pankaj Singhania et al (2022) (12)	M	3.5	ambiguous genitalia	46XX	NR5A1	–	NA	+ (3)
Kishore Shil et al (2023) (23)	M	24	small testis poor beard mustache development.	46XX	NA	+	testosterone replacement	+ (5)

NA, not available; M, male; SRY+, positive; SRY-, negative.

**Table 3:** Clinical, genetic, hormonal and therapeutic characteristics of patients with 46,XX DSD male phenotype.

Reference	Gonad	Hair distribution	Breast	Menstruation	Testosterone(nmol/L)	Estradiol(pg/mL)	FSH(mIU/ml)	LH(mIU/ml)
Ruben Lisker et al (1970) (19)	testis	N	N	–	NA	NA	NA	NA
Sarah F Slaney et al (1998) (17)	ovotestis	NA	NA	NA	NA	NA	NA	NA
Jianhong Li,Tianhua Huang et al (2004) (20)	testis(n=2)	N	NA	NA	<7.3(n=5)	NA	4.00 (3.2-6.2, n=5)	2.2 (1.5-3.1,n=5)
T. Wang et al. (2009) (15)	small testis	sparse	N	–	16.11	21.86	77.53	40.75
Xuefeng Gao et al (2012) (21)	NA	NA	NA	NA	8.07 (4.50-11.70, n=11)	28.01 (19.99-43.86, n=11)	52.67 (13.10-87.7, n=11)	22.77 (3.61-34.6, n=11)
Annalisa Vetro et al (2015) (18)	ovotestis	N	N	–	7.29	NA	53.3	19.4
Kangying Wang et al (2018) (22)	small testis	N	N	–	0.07	NA	32.12	12.61
Pankaj Singhania et al (2022) (12)	testis	NA	NA	–	<20	<20	0.95	0.10
Kishore Shil et al (2023) (23)	small testis	sparse	breast development	–	2.82	20.08	22.00	21.60

NA, not available; N, normal; -, no menstruation.

This study has several limitations. Whole-exome sequencing (WES) alone is insufficient to comprehensively dissect the genetic basis of such rare phenotypes, and a more comprehensive genomic testing strategy is recommended for subsequent similar cases to improve the diagnostic yield of etiological diagnosis. Analysis of copy number variations in key gene regions including SOX9, SOX3, RSPO1, and WNT4 would be valuable for clarifying the molecular mechanism underlying this atypical case of DSD; however, such analyses could not be performed due to constraints of the current laboratory platform and technical conditions. Furthermore, in-depth omics and functional studies, such as methylation profiling and RNA sequencing, were not conducted in the present study.

## 4 Conclusion

In summary, We reported a case of 46,XX DSD presenting with a male phenotype. Combined with clinical features, pathological confirmation of testicular tissue with seminiferous tubules, ovarian tissue with follicles, as well as definite evidence of uterus, fallopian tube structures and recurrent menstrual blood retention, the patient was diagnosed with 46,XX ovotesticular DSD with ectopic gonads and Müllerian structures, representing an extremely rare subtype of DSD. Psychological evaluation revealed that the patient had no desire for assisted reproduction and clearly wished to live as a male. However, long-term follow−up is crucial for monitoring the occurrence of gonadal tumors. After treatment, the patient successfully adapted to his social role. This study provides practical insights for the clinical diagnosis and management of such rare cases. Further clinical studies involving more similar cases and experimental research are essential to elucidate the underlying pathogenesis.

## Data Availability

The original contributions presented in the study are included in the article/supplementary material. Further inquiries can be directed to the corresponding author.
